# Relationship between corneal biomechanical parameters and corneal sublayer thickness measured by Corvis ST and UHR-OCT in keratoconus and normal eyes

**DOI:** 10.1186/s40662-020-00225-z

**Published:** 2021-01-08

**Authors:** Yong Li, Zhiqiang Xu, Qiaoli Liu, Yuzhou Wang, Kan Lin, Jiahui Xia, Shihao Chen, Liang Hu

**Affiliations:** 1 School of Ophthalmology and Optometry, Eye Hospital, Wenzhou Medical University, 270 Xueyuan road, Wenzhou, 325000 Zhejiang China; 2 National Clinical Research Center for Ocular Diseases, Wenzhou, Zhejiang China

**Keywords:** Biomechanics, Corneal thickness, Keratoconus, Corvis ST, UHR-OCT

## Abstract

**Background:**

To explore the relationship between corneal biomechanical parameters and corneal sublayer thickness using corneal visualization Scheimpflug technology (Corvis ST) and ultrahigh-resolution optical coherence tomography (UHR-OCT) in clinical and suspected keratoconus and normal eyes.

**Methods:**

Cross-sectional prospective study. A total of 94 eyes of 70 participants were recruited. Twenty five eyes of 19 keratoconus patients, 52 eyes of 34 patients showing high risk of developing keratoconus according to the Belin/Ambrosio Enhanced Ectasia Display, and each eye of 17 normal subjects were enrolled. All participants underwent Corvis ST, Pentacam, and UHR-OCT examinations at the same time. Stiffness parameter A1 (SP-A1), deformation amplitude ratio (DA ratio), and other biomechanical parameters were recorded using Corvis ST. The vertical and horizontal thickness profiles of central 3 mm corneal epithelium, Bowman’s layer, and stroma as measured by the perpendicular distance between the neighboring interfaces were generated using UHR-OCT. The flat keratometry and steep keratometry were obtained using Pentacam. Analysis of correlation was applied to explore the association between variables.

**Results:**

Most of the biomechanical parameters and corneal sublayer thickness profiles showed statistical differences among three groups. A statistically significant linear relationship was noted between SP-A1 and DA ratio in all three groups. SP-A1 was found to be positively correlated with epithelial and Bowman’s layer thickness in the keratoconus (KC) group, and with stromal thickness in all three groups. In the normal and suspected keratoconus (SKC) groups, only stromal thickness was included in the stepwise linear regression to predict SP-A1, whereas in the KC group, steep keratometry and Bowman’s layer thickness were included.

**Conclusions:**

Significant and different correlations were noted between corneal stiffness and corneal sublayer thickness in different groups, indicating that corneal sublayers may play different roles in maintaining corneal biomechanical stability between keratoconus and normal eyes.

## Background

Keratoconus (KC) is a noninflammatory disease characterized by progressive keratectasia and corneal thinning due to significant structural degeneration, finally causing severe visual impairment and acute corneal edema [1]. The diagnosis of KC is mainly focused on two aspects: corneal biomechanics and corneal imaging systems including topography and tomography.

Corneal focal biomechanical weakness is considered to play a major role during the pathological changes in KC [2–7]. Corneal visualization Scheimpflug technology (Corvis ST, Oculus Optikgeräte GmbH; Wetzlar, Germany) is commonly used to assess corneal biomechanics [8]. It provides corneal deformation indices with an ultra-high-speed Scheimpflug camera, which directly catches corneal movement under a constant metered air pulse. Stiffness parameter A1 (SP-A1) and deformation amplitude ratio (DA ratio) are two relatively novel parameters representing corneal biomechanics, which are important for KC diagnosis [2, 9] as part of preoperative examinations for refractive surgery [10].

Corneal topography is used to map the shape and features of the anterior surface of the cornea. Corneal tomography, however, evaluates the whole cornea by obtaining the corneal cross-sectional images [11]. The rapid development and application of optical coherence tomography (OCT) have made it possible to detect microstructure changes of the cornea (i.e., corneal sublayer thickness profiles), which have yielded promising results in diagnosing KC [12, 13]. Ultrahigh-resolution optical coherence tomography (UHR-OCT), with nearly 3 μm of axial resolution in corneal tissue, can provide distinct images that reveal the epithelium, Bowman’s layer, stroma, and endothelium of the cornea, allowing accurate measurements of axial thickness to verify localized changes of corneal sublayers [13, 14].

Consequently, the correlations between corneal biomechanics and corneal topography and tomography characteristics have aroused intensive research interests. Studies have demonstrated the significance of understanding the corneal epithelial profile in refractive surgery [15]. Zhao et al. reported the significant relationship between corneal stiffness and thinnest corneal thickness in KC [16]. Ziaei et al. demonstrated that corneal epithelial removal in eyes with KC undergoing cross-linking seemed to alter corneal biomechanical integrity and make the cornea more prone to deformation [17]. Seiler et al. found that Bowman’s layer does not contribute significantly to biomechanical stability within the normal cornea [18]. Moreover, it has been proven that breaks in Bowman’s layer, atypical organization of collagen fibrils, and reduced cross-linking in KC are likely to cause corneal weakness and therefore influence corneal biomechanical parameters [19, 20]. However, the potential relationship between corneal biomechanics and microstructure has not yet been described clearly.

The purpose of this study was to explore the relationship between corneal biomechanical parameters and corneal sublayer thickness in KC, suspected KC and normal eyes assessed using Corvis ST and UHR-OCT.

## Methods

### Subjects

The study was approved by the Ethics Committee of the Eye Hospital of Wenzhou Medical University Review Board. In accordance with the tenets of the Declaration of Helsinki, all subjects were recruited in the Eye Hospital of Wenzhou Medical University. Written informed consent was provided by all subjects before the study.

In this study, a total of 25 KC eyes (19 patients) were included as the keratoconus group (KC group). The diagnosis of KC was made based on the global consensus on KC [21]: at least one of the slit-lamp signs (stromal thinning, Vogt’s striae, Fleischer’s ring > 2 mm arc, or corneal scarring) along with asymmetric topographical features with inferior-superior values ≥1.9 D of the vertical gradient power across the 6 mm region. Fifty two eyes (34 patients) with a high risk of developing KC were defined as the suspected keratoconus group (SKC group), which met the following criteria: best corrected visual acuity (BCVA) ≥ 1.0, normal-appearing cornea on slit-lamp biomicroscopy and ophthalmoscopy, having red (at least 2.6 standard deviation from the mean) or yellow (at least 1.6 standard deviation from the mean) color-coded number in at least one of the five differential parameters (Df, Db, Dp, Dt, and Da) in the Belin/Ambrosio Enhanced Ectasia Display (BAD) with white or yellow coded number in the final parameter “D” [22, 23]. Since KC affects both eyes in one patient unequally, all eyes that met the above criteria were included. Seventeen healthy subjects were included in the normal control group with normal quantitative parameters and patterns in Pentacam and slit-lamp examinations, and only the right eyes were analyzed. Eyes with a history of any previous ocular surgery, corneal scar or inflammation, any episodes of corneal edema, or other ocular diseases, wearing rigid gas permeable (RGP) lenses within 4 weeks or soft contact lenses within 2 weeks were excluded.

**Fig. 1:**
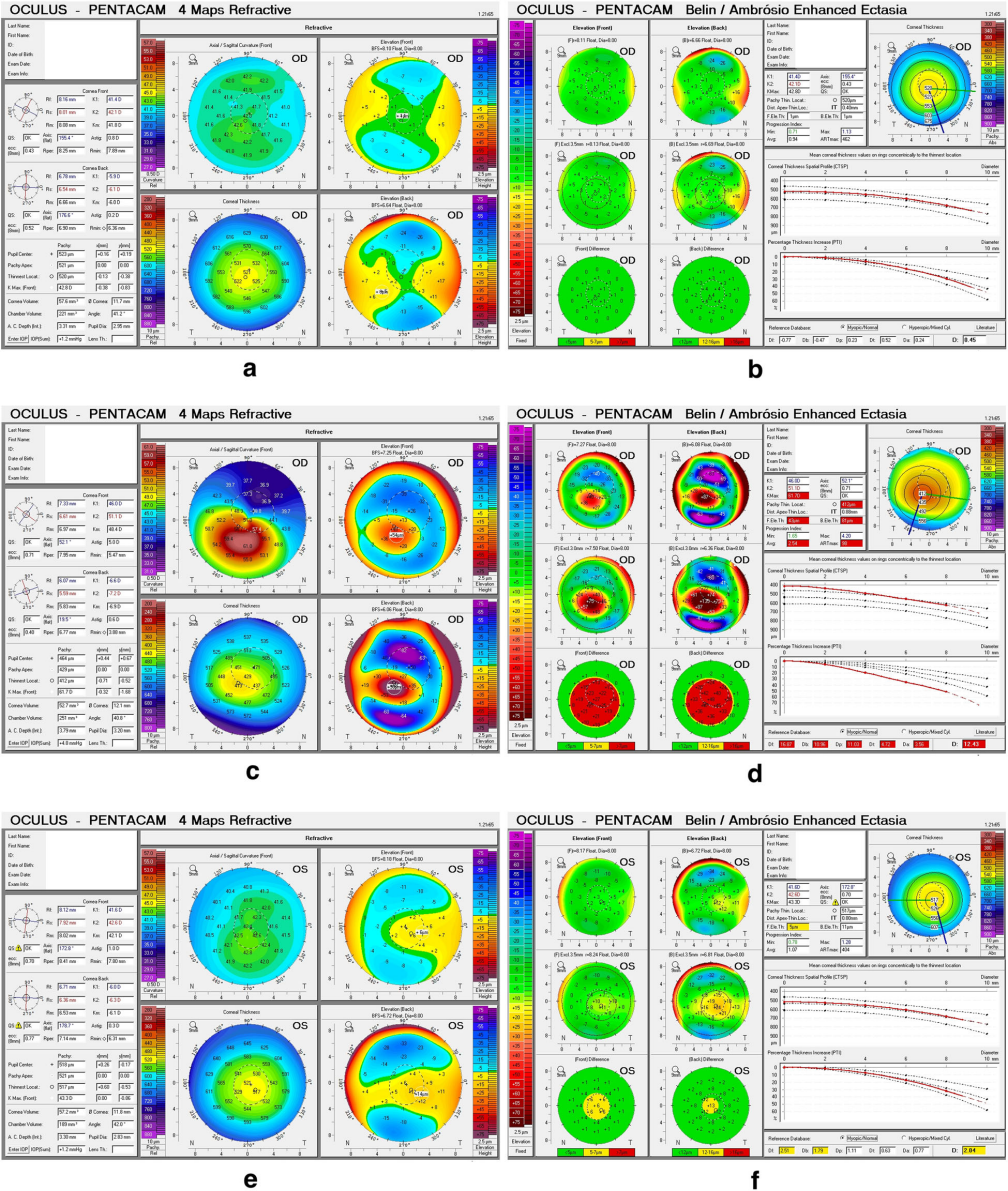
Sample topography images of each group. **a** 4 Maps Refractive of a normal subject. **b** BAD of a normal subject. **c** 4 Maps Refractive of a KC subject. **d** BAD of a KC subject. **e** 4 Maps Refractive of a SKC subject. **f** BAD of a SKC subject. BAD, Belin/Ambrosio Enhanced Ectasia Display; KC, keratoconus; SKC, suspected keratoconus

Each patient underwent comprehensive ocular examinations at the same time between 9 am and 5 pm by the same operator (QL), including uncorrected and best corrected visual acuity, manifest refraction, slit-lamp biomicroscopy examination, examinations using the Pentacam, Corvis ST, and UHR-OCT instruments. The sample topography images for each group are presented in Fig. 1.

### Experimental procedure

The Corvis ST (Corvis ST, Oculus Optikgeräte GmbH; Wetzlar, Germany) provides information concerning corneal response to a constant air pulse by emitting a quick, controlled air impulse to deform the cornea. To avoid miscalculations of poor imaging quality, the measurement quality is displayed in a specific QS (Quality Specification) window. Only results with “OK” in the QS window indicating good image quality were included in the statistical analyses. Each eye underwent examination three times to obtain a mean value. The following parameters were recorded: stiffness parameter A1 (SP-A1, 24], corneal maximum ingoing velocity at first applanation (A1V), corneal maximum outgoing velocity at second applanation (A2V), distance between the two peaks of the cornea at highest concavity (PD), displacement of corneal apex at highest concavity in reference to initial state (HCDfA), ratio of deformation amplitude at corneal apex to deformation amplitude at points 2-mm peripheral to apex at highest concavity (DA ratio), radius of curvature at highest concavity (HCR), integrated radius (IR) [24], the Ambrosio relational thickness to the horizontal profile (ARTh), the Tomographic and Biomechanical Index (TBI), and the Corvis Biomechanical Index (CBI).

**Fig. 2:**
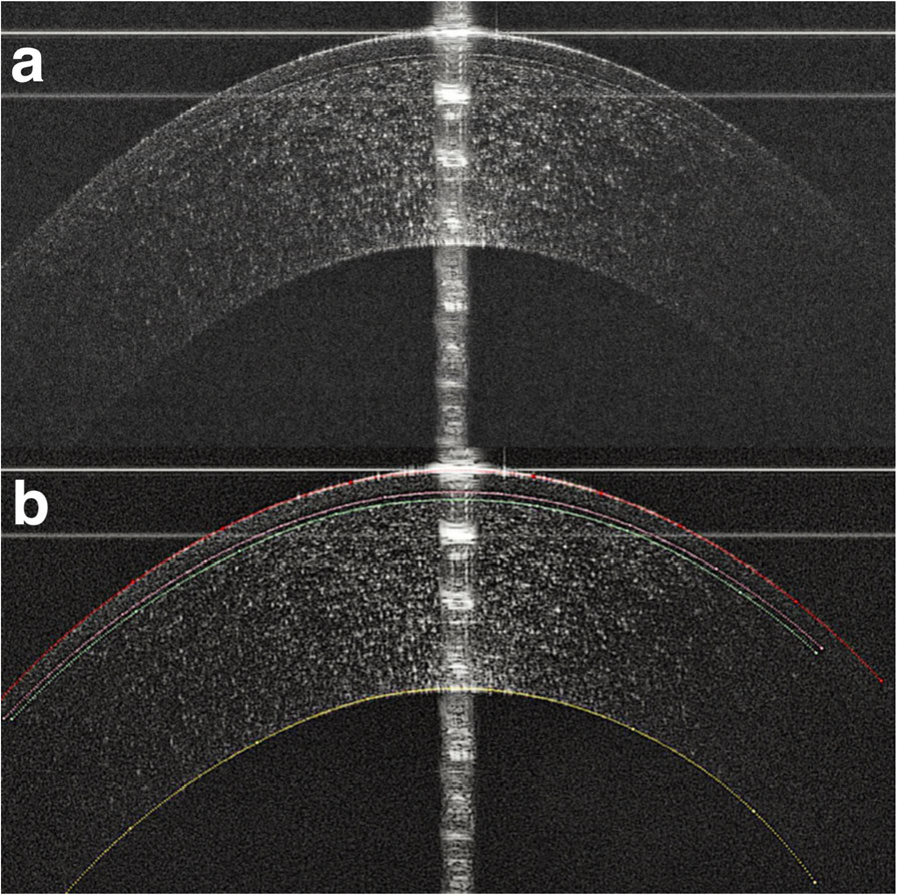
Using J-OCT to obtain the corneal thickness profile in a normal eye. **a** The original image of the central horizontal meridian of one normal eye. A specular reflection of the corneal apex ensured that the OCT scanning probe was aligned perpendicular to the cornea. **b** The image processed by the J-OCT. The red line corresponds to the outer surface of the epithelium; the pink line corresponds to the inner surface of the epithelium; the green line corresponds to the inner surface of the Bowman’s layer; and the yellow line corresponds to the endothelial layer. Stromal thickness was measured as the perpendicular distance between the inner surface of Bowman’s layer and the endothelial layer, which include the stroma, Descemet’s membrane, and the endothelium biologically. The latter two layers were too thin to be noted in the image

Each patient underwent imaging using a custom-built UHR-OCT with 3 μm of axial resolution in corneal tissue, which has been described previously [14, 25–28]. The image was acquired with a speed of 24 k A-line per second and B scan comprised of 1365 × 2048 pixels, equal to a scan depth of 2.02 mm and a width of 8.66 mm in the air. The patients were required to look straight ahead to image the central vertical and horizontal cornea. The measurements of both directions were performed three times by the same experienced operator. The central cornea in the vertical and horizontal 3 mm zone were analyzed using a custom software (J-OCT-1, version 1.0) to produce the thickness profiles of corneal epithelium, Bowman’s layer, and stroma as measured by the perpendicular distance between the neighboring interfaces (Fig. 2) at 0.5 mm steps with an average matrix [27, 28]. A custom algorithm according to Snell’s principle was used to eliminate the distortion of images caused by refraction and transition of the group index. A refraction index of 1.389 was used.

All participants underwent examination using Pentacam (Oculus Optikgeräte GmbH, Wetzlar, Germany). Only results with “OK” in the QS window were recorded to ensure the imaging quality (Fig. 1). Each eye underwent examination three times to obtain a mean value. The color of the parameters in the BAD was one of the important enrollment criteria that defines the SKC group. The flat keratometry and steep keratometry of the cornea were recorded.

### Statistical analysis

The continuous variables results are presented as mean ± standard deviation (SD). The normality of all variables was identified by histogram and the Shapiro-Wilk test. To determine the differences among the three groups, the normally distributed variables were compared using the one-way analysis of variance (ANOVA) with the Tukey or Games-Howell post-hoc tests; otherwise, the Kruskal-Wallis test was used. The Pearson or Spearman correlation test was applied to determine the correlation between parameters. Partial correlation test was applied subsequently to determine the amount of variance in the dependent variable uniquely explained by the independent variable after adjusting other covariates. It was used to determine the correlation between Corvis ST-acquired parameters and the thickness of one single corneal sublayer controlling for the other two layers. Stepwise multiple linear regression analysis was performed to assess the effect of the independent variables on SP-A1. All statistical analyses of the study were performed with IBM SPSS version 23.0 (SPSS for Mac, Inc., Chicago, IL, USA). *P* < 0.05 indicates a statistically significant difference.

## Results

The demographics and all the measured parameters of the three groups are presented in Table 1. Overall, the mean age and gender ratio were not significantly different among groups. SP-A1 was 107.05 ± 15.84, 71.67 ± 17.34, 99.49 ± 12.66 in the normal, KC, and SKC group, respectively. DA ratio was 4.37 ± 0.37, 5.55 ± 0.85, 4.47 ± 0.43 in the normal, KC, and SKC group, respectively. SP-A1, DA ratio, HCR, IR, ARTh, TBI, CBI, and all three sublayers thickness were statistically different between the KC and normal groups. Additionally, ARTh, TBI, and Bowman’s layer thickness showed statistically significant differences between the SKC and normal groups.

**Table 1:** Intergroup differences of all measured parameters among normal, KC, and suspected KC groups

Parameter	Mean ± SD			ANOVA	Normal vs. KC P	Normal vs. SKC P
	Normal (*n* = 17)	KC (*n* = 25)	SKC (*n* = 52)			
Age (years)	23.44 ± 2.00	25.11 ± 7.36	23.70 ± 5.85	0.629	0.63	0.972
Gender (M/F)	10/7	11/8	14/20	–	–	–
Cylinder (D)	−0.65 ± 0.56	−3.41 ± 2.29	−0.77 ± 0.61	0.000^a^	0.000^a^	0.933
SE (D)	−3.98 ± 3.41	−4.25 ± 2.89	−4.97 ± 2.02	0.280	0.944	0.353
A1V (m/s)	0.15 ± 0.02	0.16 ± 0.03	0.15 ± 0.02	0.021^a^	0.213	0.841
A2V (m/s)	−0.38 ± 0.04	−0.352 ± 0.09	−0.34 ± 0.04	0.069	0.244	0.055
PD (mm)	4.90 ± 0.22	4.82 ± 0.23	4.82 ± 0.24	0.428	0.541	0.409
HCR (mm)	6.81 ± 0.60	5.36 ± 0.76	6.64 ± 0.52	0.000^a^	0.000^a^	0.550
HCDfA (mm)	1.10 ± 0.10	1.14 ± 0.12	1.07 ± 0.09	0.018^a^	0.430	0.508
SP-A1	107.05 ± 15.84	71.67 ± 17.34	99.49 ± 12.66	0.000^a^	0.000^a^	0.203
IR	8.78 ± 0.88	12.41 ± 2.43	9.38 ± 0.90	0.000^a^	0.000^a^	0.316
ARTh	412.88 ± 64.94	206.41 ± 80.57	363.70 ± 50.89	0.000^a^	0.000^a^	0.016^a^
DA ratio	4.37 ± 0.37	5.55 ± 0.85	4.47 ± 0.43	0.000^a^	0.000^a^	0.816
TBI^b^	0.17 ± 0.15	1.00	0.49 ± 0.28	–	0.000^a^	0.004^a^
CBI^b^	0.13 ± 0.18	0.99 ± 0.04	0.23 ± 0.30	–	0.000^a^	1.000
Flat K (D)^b^	42.52 ± 0.97	45.27 ± 2.47	43.20 ± 1.34	–	0.000^a^	0.019^a^
Steep K (D)	43.56 ± 1.29	48.67 ± 3.43	44.41 ± 1.43	0.000^a^	0.000^a^	0.015^a^
EPT (μm)	53.53 ± 2.00	48.17 ± 3.16	53.52 ± 2.26	0.000^a^	0.000^a^	0.936
BLT (μm)	17.96 ± 1.58	15.69 ± 1.32	16.12 ± 1.19	0.000^a^	0.000^a^	0.000^a^
STT (μm)	480.65 ± 32.01	414.10 ± 29.43	460.57 ± 33.89	0.000^a^	0.000^a^	0.074

*Normal* = normal group; *KC* = keratoconus group; *SKC* = suspected keratoconus group; *n* = number of eyes; *Cylinder* = cylindrical power; *SE* = spherical equivalent; *A1V* = corneal apex velocity at first applanation; *A2V* = corneal apex velocity at second applanation; *PD* = peak distance; *HCR* = highest concavity radius of curvature; *HCDfA* = deflection amplitude at highest concavity; *SP-A1* = stiffness parameter at first applanation; *IR* = integrated radius; *ARTh* = Ambrosio relational thickness to the horizontal profile; *DA* = deformation amplitude; *TBI* = tomographic and biomechanical index; *CBI* = Corvis biomechanical index; *Flat K* = flat keratometry; *Steep K* = steep keratometry; *EPT* = epithelial thickness; *BLT* = Bowman’s layer thickness; *STT* = stromal thickness; *D* = diopter; ^a^*P* < 0.05; ^b^Kruskal-Wallis test

**Fig. 3:**
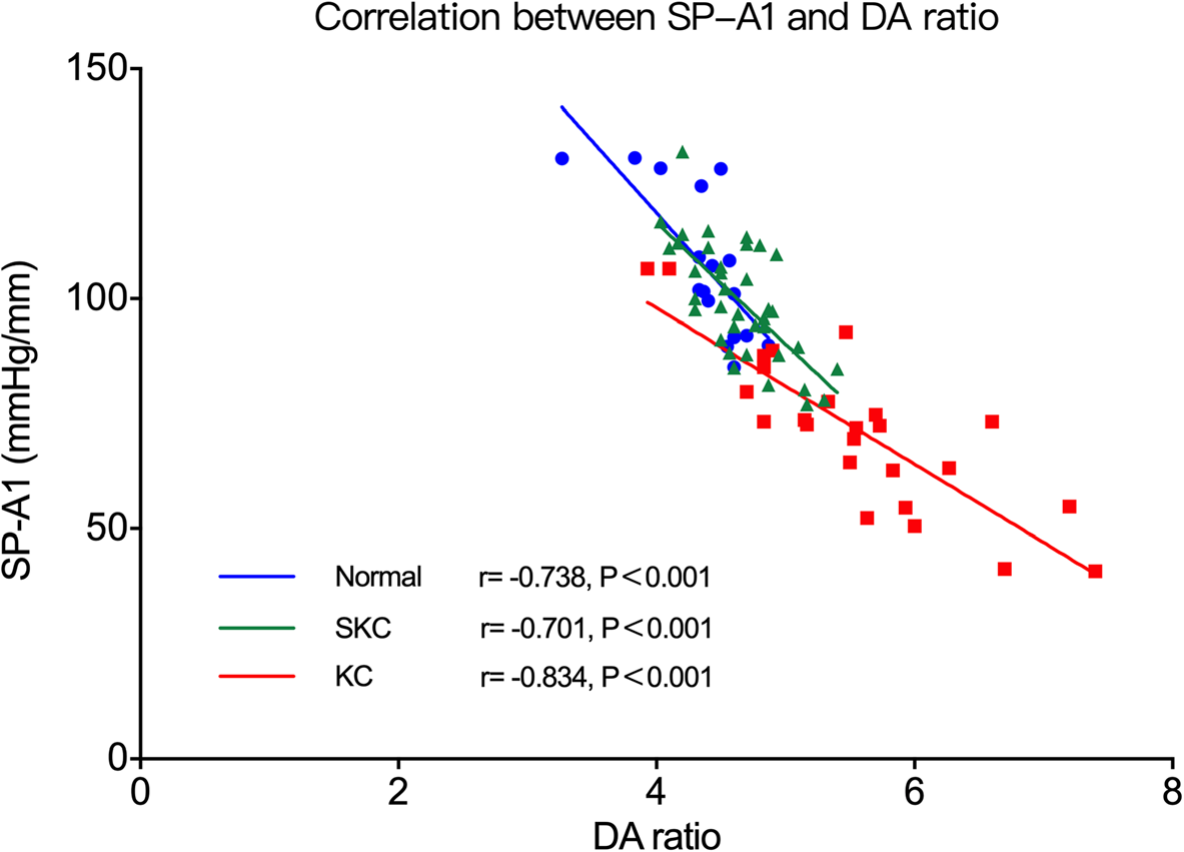
Correlation between SP-A1 and DA ratio. SP-A1 was significantly negatively correlated with DA ratio in all three groups. Normal, normal group; KC, keratoconus group; SKC, suspected keratoconus group

SP-A1 was found to have a negative correlation with steep K in the KC group (*r* = −0.690, *P* < 0.001), but no correlation in the normal or SKC groups. In addition, the correlations between SP-A1 and other Corvis ST-acquired parameters were analyzed. The most noteworthy finding was the significant negative correlation between SP-A1 and DA ratio (normal group: *r* = −0.738, *P* < 0.001; KC group: *r* = −0.834, *P* < 0.001; SKC group: *r* = −0.701, *P* < 0.001) (Fig. 3).

**Fig. 4:**
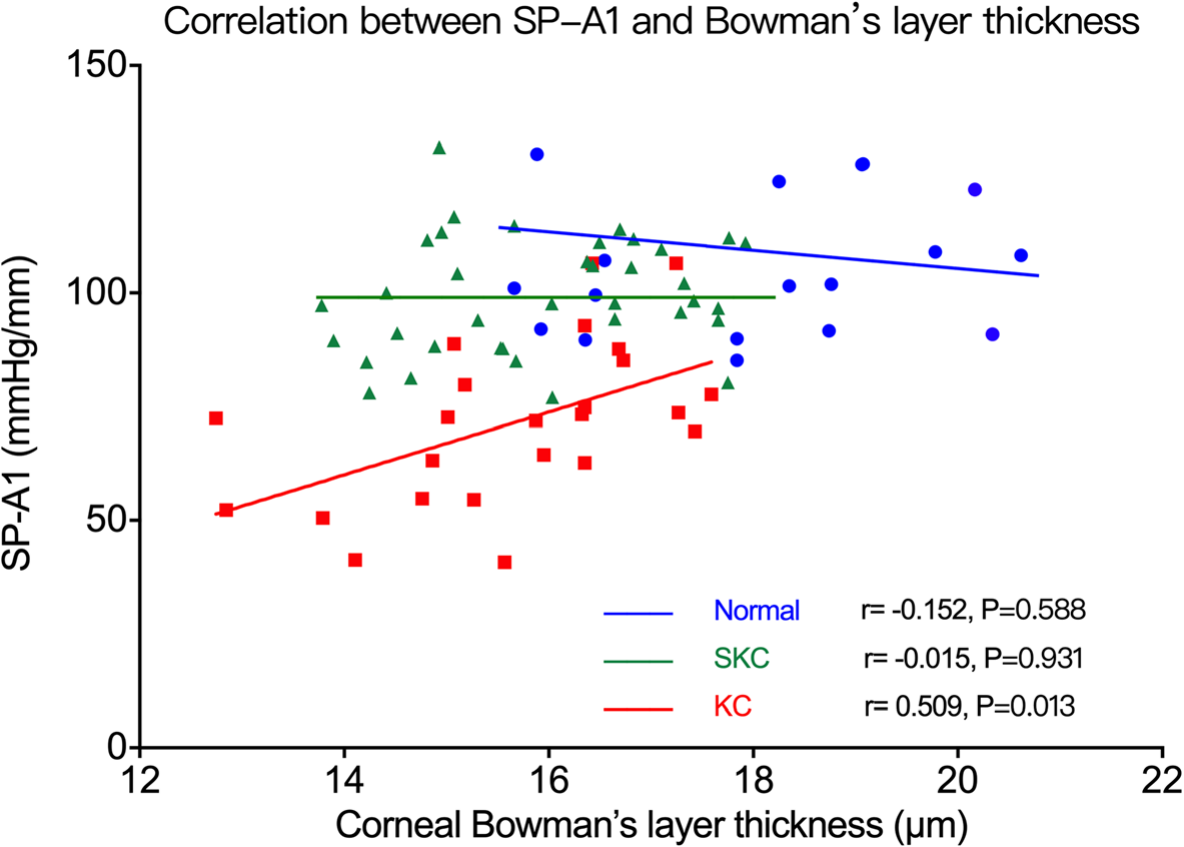
Correlation between SP-A1 and Bowman’s layer thickness. SP-A1 was positively correlated with Bowman’s layer thickness in the KC group. Normal, normal group; KC, keratoconus group; SKC, suspected keratoconus group

**Fig. 5:**
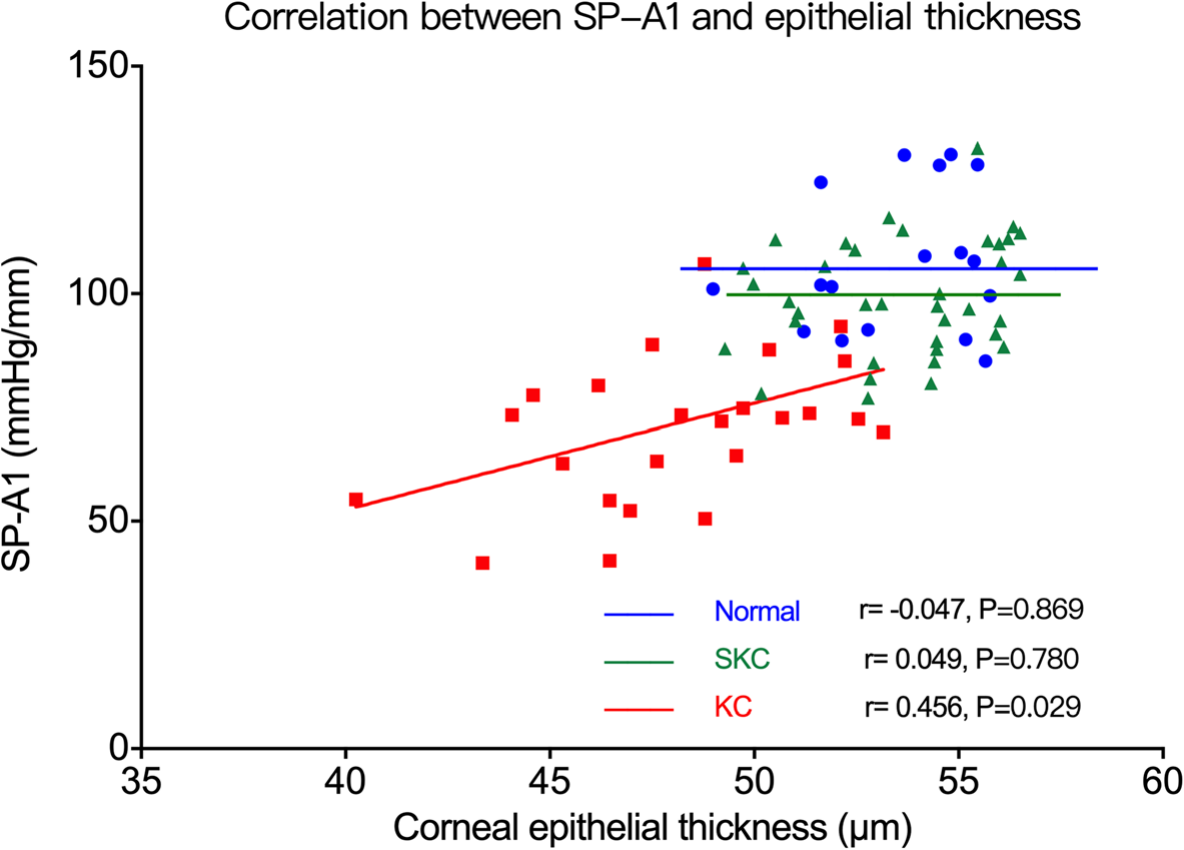
Correlation between SP-A1 and epithelial thickness. SP-A1 was positively correlated with epithelial thickness in the KC group. Normal, normal group; KC, keratoconus group; SKC, suspected keratoconus group

**Fig. 6:**
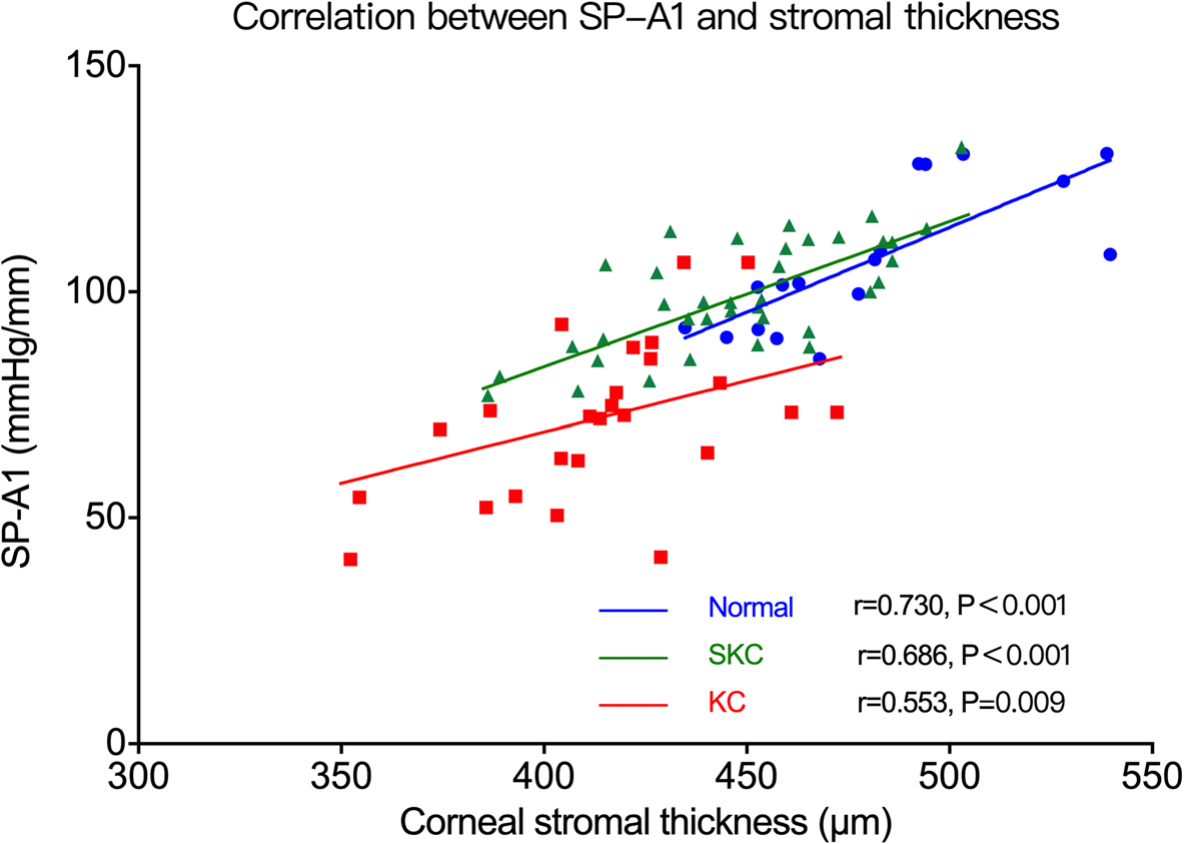
Correlation between SP-A1 and stromal thickness. SP-A1 was positively correlated with stromal thickness in all three groups. Normal, normal group; KC, keratoconus group; SKC, suspected keratoconus group

The partial correlations between biomechanical parameters and sublayer thickness are listed in Tables 2, 3 and 4. SP-A1 was positively correlated with Bowman’s layer thickness (*r* = 0.509, *P* = 0.013; Fig. 4) and epithelial thickness (*r* = 0.456, *P* = 0.029; Fig. 5) in the KC group, and with stromal thickness in all three groups (normal group: *r* = 0.730, *P* < 0.001; KC group: *r* = 0.533, *P* = 0.009; SKC group: *r* = 0.686, *P* < 0.001) (Fig. 6). A1V was negatively correlated with Bowman’s layer thickness in the KC group (*r* = −0.574, *P* = 0.003) and with stromal thickness in the KC and SKC groups. In the KC group, A2V was found to be positively correlated with Bowman’s layer and stromal thickness. Moreover, HCR showed a positive correlation with stromal thickness in the SKC group (*r* = 0.386, *P* = 0.005), but with epithelial thickness in the KC group (*r* = 0.447, *P* = 0.025). IR and DA ratio were negatively correlated with stromal thickness in all three groups, and with epithelial thickness in the KC group. In addition, CBI was negatively correlated with stromal thickness in the normal and SKC groups, while TBI showed no correlation with any thickness parameters in three groups.

**Table 2:** Correlations between biomechanical parameters and corneal sublayer thickness in the normal group

		EPT	BLT	STT
SP-A1^a^	r	0.184	0.330	0.760
	P	0.479	0.195	0.000^d^
SP-A1^c^	r	−0.047	−0.152	0.730
	P	0.869	0.588	0.000^d^
A1V^a^	r	0.016	−0.034	−0.381
	P	0.952	0.897	0.131
A2V^a^	r	−0.082	−0.168	0.116
	P	0.753	0.519	0.656
PD^a^	r	0.051	−0.008	−0.371
	P	0.845	0.974	0.143
HCR^a^	r	0.088	0.079	0.466
	P	0.736	0.762	0.059
HCDfA^a^	r	0.055	−0.008	−0.275
	P	0.835	0.974	0.285
IR^a^	r	−0.155	−0.059	−0.549
	P	0.552	0.821	0.023^d^
ARTh^a^	r	0.264	−0.010	0.465
	P	0.307	0.970	0.060
DA ratio^a^	r	−0.149	−0.025	−0.523
	P	0.568	0.924	0.031^d^
CBI^b^	r	−0.200	−0.088	−0.519
	P	0.442	0.738	0.033^d^
TBI^b^	r	−0.159	−0.382	−0.178
	P	0.541	0.131	0.495

*EPT* = epithelial thickness; *BLT* = Bowman’s layer thickness; *STT* = stromal thickness; *SP-A1* = stiffness parameter at first applanation; *A1V* = corneal apex velocity at first applanation; *A2V* = corneal apex velocity at second applanation; *PD* = peak distance; *HCR* = highest concavity radius of curvature; *HCDfA* = deflection amplitude at highest concavity; *IR* = integrated radius; *ARTh* = Ambrosio relational thickness to the horizontal profile; *DA* = deformation amplitude; *CBI* = Corvis biomechanical index; *TBI* = tomographic and biomechanical index; ^a^Pearson correlation test; ^b^Spearman correlation test; ^c^Partial correlation analysis controlling for the thickness of the other two layers; ^d^*P* < 0.05

**Table 3:** Correlations between biomechanical parameters and corneal sublayer thickness in the KC group

		EPT	BLT	STT
SP-A1^a^	r	0.427	0.527	0.515
	P	0.033^d^	0.007^d^	0.008^d^
SP-A1^c^	r	0.456	0.509	0.533
	P	0.029^d^	0.013^d^	0.009^d^
A1V^a^	r	−0.355	−0.574	−0.495
	P	0.082	0.003^d^	0.012^d^
A2V^a^	r	0.028	0.488	0.676
	P	0.895	0.013^d^	0.000^d^
PD^a^	r	−0.102	−0.431	−0.351
	P	0.627	0.031^d^	0.085
HCR^a^	r	0.447	0.276	0.150
	P	0.025*	0.181	0.474
HCDfA^a^	r	−0.346	−0.495	−0.355
	P	0.090	0.012^d^	0.081
IR^b^	r	−0.483	−0.495	−0.362
	P	0.015^d^	0.012^d^	0.075
ARTh^a^	r	0.292	0.130	0.560
	P	0.157	0.537	0.004^d^
DA ratio^a^	r	−0.544	−0.390	−0.484
	P	0.005^d^	0.054	0.014^d^
CBI^b^	r	−0.145	0.005	−0.325
	P	0.488	0.981	0.112
TBI	r	–	–	–
	P	–	–	–

*EPT* = epithelial thickness; *BLT* = Bowman’s layer thickness; *STT* = stromal thickness; *SP-A1* = stiffness parameter at first applanation; *A1V* = corneal apex velocity at first applanation; *A2V* = corneal apex velocity at second applanation; *PD* = peak distance; *HCR* = highest concavity radius of curvature; *HCDfA* = deflection amplitude at highest concavity; *IR* = integrated radius; *ARTh* = Ambrosio relational thickness to the horizontal profile; *DA* = deformation amplitude; *CBI* = Corvis biomechanical index; *TBI* = tomographic and biomechanical index; ^a^Pearson correlation test; ^b^Spearman correlation test; ^c^Partial correlation analysis controlling for the thickness of the other two layers; ^d^*P* < 0.05

**Table 4:** Correlations between biomechanical parameters and corneal sublayer thickness in the suspected KC (SKC) group

		EPT	BLT	STT
SP-A1^a^	r	0.223	0.186	0.721
	P	0.185	0.270	0.000^d^
SP-A1^c^	r	0.049	−0.015	0.686
	P	0.780	0.931	0.000^d^
A1V^a^	r	−0.057	−0.141	−0.421
	P	0.691	0.320	0.002^d^
A2V^a^	r	−0.059	0.214	0.353
	P	0.677	0.128	0.010^d^
PD^a^	r	−0.159	−0.338	−0.413
	P	0.260	0.014^d^	0.002^d^
HCR^a^	r	−0.020	0.224	0.386
	P	0.887	0.111	0.005^d^
HCDfA^a^	r	−0.007	−0.301	−0.365
	P	0.963	0.030^d^	0.008^d^
IR^a^	r	−0.054	0.258	−0.654
	P	0.702	0.064	0.000^d^
ARTh^a^	r	0.162	0.095	0.242
	P	0.252	0.502	0.084
DA ratio^a^	r	−0.035	−0.373	−0.815
	P	0.803	0.006^d^	0.000^d^
CBI^b^	r	−0.175	−0.268	−0.644
	P	0.214	0.055	0.000^d^
TBI^b^	r	0.143	−0.227	−0.116
	P	0.313	0.106	0.414

*EPT* = epithelial thickness; *BLT* = Bowman’s layer thickness; *STT* = stromal thickness; *SP-A1* = stiffness parameter at first applanation; *A1V* = corneal apex velocity at first applanation; *A2V* = corneal apex velocity at second applanation; *PD* = peak distance; *HCR* = highest concavity radius of curvature; *HCDfA* = deflection amplitude at highest concavity; *IR* = integrated radius; *ARTh* = Ambrosio relational thickness to the horizontal profile; *DA* = deformation amplitude; *CBI* = Corvis biomechanical index; *TBI* = tomographic and biomechanical index; ^a^Pearson correlation test; ^b^Spearman correlation test; ^c^Partial correlation analysis controlling for the thickness of the other two layers; ^d^*P* < 0.05

The results of stepwise multiple linear regression model analysis are presented in Table 5. For both normal and SKC groups, only stromal thickness was included in the regression equation to predict SP-A1; whereas for the KC group, the steep K and Bowman’s layer thickness were included (BLT, Bowman’s layer thickness; STT, stromal thickness):

- SP-A1 = − 73.731 + 0.376STT (normal group);
- SP-A1 = 138.830–3.077steep K + 5.266BLT (KC group);
- SP-A1 = − 41.086 + 0.313STT (SKC group).

**Table 5:** Stepwise multiple linear regression model analysis for predicting theoretical SP-A1 in three groups

Groups	Main predictors	B	SE	β	t	P	Adjusted R^2^	F	P
Normal	Constant	−73.731	40.029		−1.842	0.085	0.577	20.481	0.000
	STT	0.376	0.083	0.760	4.526	0.000			
KC group	Constant	138.830	46.527		2.984	0.007	0.598	18.854	0.000
	Steep K	−3.077	0.669	−0.608	−4.599	0.000			
	BLT	5.266	1.731	0.402	3.042	0.006			
SKC group	Constant	−76.349	20.140		−3.791	0.000	0.619	83.792	0.000
	STT	0.399	0.044	0.791	9.154	0.000			

*Normal* = normal group; *KC* = keratoconus group; *SKC* = suspected keratoconus group; *B* = unstandardized coefficients; *SE* = standard error of unstandardized coefficients; *β* = standardized coefficients (beta); *t* = unstandardized coefficient/standard error; *STT* = stromal thickness; *BLT* = Bowman’s layer thickness; *Steep K* = steep keratometry

## Discussion

Studies have demonstrated that the alteration in corneal biomechanical properties play a significant role in the generation and progression of KC [20]. The Corvis ST provides several dynamic corneal response parameters within different phases of corneal deformation. SP-A1 is a novel stiffness parameter to quantify corneal resistance to deformation defined as the ratio of the pressure loading on the cornea to the displacement between the apex of the undeformed cornea and the deflection at first applanation [9, 29, 30]. It is a valuable parameter representing corneal stiffness and intrinsic biomechanics that takes into account confounding factors such as intraocular pressure and eye movement. DA ratio is another new parameter defined as the deformed amplitude of the central apex divided by the average deformation of two points located 2 mm on either side of the apex [29]. The ratios are expected to be higher in ectatic corneas, which are less resistant to deformation. Some studies have identified the superiority of DA ratio among all the dynamic corneal response parameters in differentiating KC [9, 31]. CBI is based on linear regression analysis of dynamic corneal response parameters in combination with corneal horizontal thickness profile [30], while TBI is based on a combination of biomechanical and tomographic data from the Corvis ST and Pentacam, along with artificial intelligence optimization [32].

In the present study, a partial correlation test analysis was performed to determine the amount of variance in corneal biomechanics uniquely explained by the corneal single sublayer thickness, excluding the confounding effect of the other two layers. We found significant correlations between corneal stiffness and sublayer thickness in different groups, indicating that corneal sublayers may contribute differently to biomechanical stability between KC and normal eyes.

SP-A1 was positively correlated with stromal thickness in all three groups, and the correlation coefficient decreased from normal to KC eyes. Interestingly, in the KC group, we found positive correlations between SP-A1 and epithelial, Bowman’s layer thickness. However, no such correlation was found in the normal or SKC groups. The hypothesis of KC etiology indicates that the loss of corneal structural integrity triggers the weakness of biomechanical properties, which causes focal weakening in the cornea [33, 34]. Under the same intraocular pressure, the focal area tends to strain to a greater extent than the other area, which leads to cornea focal thinning, further deteriorating the biomechanical properties, and further thinning. Our results also supports this hypothesis. Ziaei et al. compared the biomechanical parameters of the cornea after epithelial removal in eyes with KC undergoing corneal cross-linking and suggested that corneal epithelium may play a more significant role in corneal biomechanical properties in patients with KC, which was in line with our results [17].

Previous studies have demonstrated that the corneal stroma mainly consists of collagen lamellae and accounts for nearly 90% of the total thickness of the cornea [20]. The majority of the stiffness arises from layers of collagen lamellae, which play a dominant role in corneal biomechanical support [35]. Therefore, the thickness of the stroma layer is expected to be positively correlated with corneal stiffness. For evident KC, pathological changes such as atypical organization of lamellae structure of collagen fibers, and distinct reduction of cross-links in stroma may decrease the contribution of corneal stroma to stiffness [19]. It can be presumed that the corneal epithelium and Bowman’s layer tend to compensate for deteriorating corneal biomechanical stability in KC eyes. This may underlie the correlations between corneal stiffness, epithelial and Bowman’s layer thickness in the KC group.

It is worth noting that no significant correlation was found between any biomechanical parameters and epithelial or Bowman’s layer thickness in the normal or SKC groups. This indicates that in a healthy cornea or one at the very early stage of KC, the epithelium and Bowman’s layer play only limited roles in maintaining biomechanical stability. In a transepithelial photorefractive keratectomy (PRK), ablation of the corneal epithelium and stroma is performed, during which Bowman’s layer is destroyed [36]. Studies have demonstrated that no harmful effects of removal of Bowman’s layer over the central cornea have been noted in patients who have had PRK [37]. Based on the results of our study, it can be inferred that damaging the Bowman’s layer during transepithelial PRK surgery will not influence biomechanical stability in a healthy cornea. However, preoperative examinations to exclude any risk of developing corneal ectatic diseases such as KC cannot be ignored [38, 39].

Studies have shown that corneal keratometry can frequently affect corneal response parameter measurements [40], which may underlie the negative correlation between steep K and SP-A1 in the KC group. Interestingly, the results of stepwise multiple linear regression analysis of KC group included steep K and Bowman’s layer thickness to predict SP-A1. Our finding was consistent even when controlling for steep K and flat K in partial correlation analysis. It can be assumed that in KC patients, the effect of corneal epithelium and stroma on corneal stiffness can be interpreted as correlation with corneal keratometry alteration.

In this study, we found a significant decrease of SP-A1 and increase of DA ratio in the KC group compared with the normal group. According to a recent review, there is no consensus on what features are relevant to diagnose the early form of KC [23]. In our study, slight changes of the two indices can be noticed in the SKC group, which indicated an early change of biomechanical properties in the progression of KC. In addition, significant negative correlations between SP-A1 and DA ratio were identified in all three groups. It can be concluded that the lower the corneal stiffness, the less resistant to deformation and, therefore, the greater the DA ratio. Likewise, Sedaghat et al. [41] assessed biomechanical parameters in 145 eyes with frank KC and reported the diagnostic efficacy of SP-A1 (AUC = 0.965) and DA ratio (AUC = 0.950) in detecting frank KC. Herber et al. [40] also compared Corvis ST-acquired data in KC and normal eyes, and found that SP-A1 and DA ratio had higher diagnostic efficacy in differentiating KC versus other parameters.

When analyzing UHR-OCT-generated thickness profiles of the epithelium, Bowman’s layer, and stroma, we found significant thinning of all three layers in KC eyes compared with normal eyes. Bowman’s layer thinning also occurred in the SKC group, which suggested that the alteration of Bowman’s layer might take place during the early progression of KC. Previous studies have demonstrated the change in the lamellar structure of Bowman’s layer collagen fibers during KC progression, which may provide an explanation for our results [13, 14].

This is the first study to demonstrate the correlations between corneal biomechanical parameters acquired by the Corvis ST and corneal microstructure generated by UHR-OCT. The limitations of our study are as follows. First, the sample size may have limited the generalization of our results. Second, since participants were included in different groups partly according to Pentacam imaging results, corneal keratometry could be an influencing factor for analyses. Multi-center studies containing larger sample sizes with different stages of KC according to corneal keratometry are warranted to further explore corneal thickness and keratometry alteration during KC progression and their combined effects on corneal biomechanics. Third, the confounding effect of intraocular pressure (IOP) on corneal response and the measurement of corneal biomechanical parameters cannot be ignored. Recently, Eliasy et al. had reported a new stiffness parameter (Stress-Strain Index or SSI) that showed no significant correlation with both central corneal thickness and IOP [42]. In short, further studies with follow-up examinations to validate the present results, and studies using SSI as the main biomechanical parameter to exclude the effect of IOP are warranted.

## Conclusions

In conclusion, we found significant and different correlations between corneal stiffness and corneal microstructure in different groups, indicating that corneal sublayers may play different roles in maintaining corneal biomechanics between keratoconus and normal eyes. Comprehensive preoperative examinations to exclude the risk of developing KC are needed to ensure the safety of refractive surgery.

## Data Availability

The datasets used and analyzed for the present study are available from the corresponding authors upon reasonable request.
